# Testicular Tumors and Environmental Pollution: A Comparative Oncoepidemiology Study in the Campania Region from 2020 to 2023

**DOI:** 10.3390/vetsci12080695

**Published:** 2025-07-24

**Authors:** Evaristo Di Napoli, Davide De Biase, Barbara degli Uberti, Maria Dimatteo, Loredana Baldi, Stefania Cavallo, Guido Rosato, Daniela Izzillo, Giuseppe Piegari, Valeria Russo, Sabrina Rossetti, Francesca Bruzzese, Chiara Palmieri, Alfredo Budillon, Orlando Paciello

**Affiliations:** 1 Department of Veterinary Medicine and Animal Production, University of Naples Federico II, 80137 Naples, Italy; giuseppe.piegari@unina.it (G.P.); valeria.russo@unina.it (V.R.); paciello@unina.it (O.P.); 2 Department of Pharmacy, University of Salerno, 84084 Fisciano, Italy; ddebiase@unisa.it; 3 Istituto Zooprofilattico Sperimentale del Mezzogiorno, 80055 Portici, Italy; barbara.degliuberti@izsmportici.it (B.d.U.); maria.dimatteo@izsmportici.it (M.D.); loredana.baldi@izsmportici.it (L.B.); stefania.cavallo@izsmportici.it (S.C.); 4 ASL Napoli 1 Centro-CRIUV Region Campania, 80145 Naples, Italy; guido.rosato@aslnapoli1centro.it (G.R.); daniela.izzillo@aslnapoli1centro.it (D.I.); 5 Uro-Gynecological Oncology, Istituto Nazionale Tumori—IRCCS—Fondazione G. Pascale, 80131 Naples, Italy; s.rossetti@istitutotumori.na.it; 6 Experimental Animal Unit, Istituto Nazionale Tumori—IRCCS—Fondazione G. Pascale, 80131 Naples, Italy; f.bruzzese@istitutotumori.na.it; 7 School of Veterinary Science, The University of Queensland, Gatton, QLD 4343, Australia; c.palmieri@uq.edu.au; 8 Scientific Directorate, Istituto Nazionale Tumori—IRCCS—Fondazione G. Pascale, 80131 Naples, Italy; a.budillon@istitutotumori.na.it

**Keywords:** cancer risk, endocrine disruptors, comparative oncology, animal cancer registry, data science, One Health

## Abstract

A comparative analysis of testicular tumors in dogs and humans highlights notable similarities in cancer behavior, particularly regarding environmental influences. The ubiquitous distribution of endocrine disruptors in industrialized countries and their effects are strongly linked to the development of testicular cancers. This study examined 221 and 174 cases of testicular tumor in dogs and humans, respectively, from the Campania Animal Cancer Registry and the database of the National Cancer Institute “Fondazione G. Pascale-IRCCS”, between 2020 and 2023. These data were integrated with environmental data from the Ministry of Environment and Energy Security website and the Campania Regional Agency for Environmental Protection (ARPAC). The areas with the highest frequency of testicular tumors in both species were the municipalities of Napoli, Salerno, and Caserta, and their corresponding provinces. The same locations experienced severe contamination of soil and water by environmental pollutants, probably correlated to testicular tumors. Although the molecular mechanisms are not yet fully understood, our data suggest a strong link between the frequency of testicular tumors in humans and dogs and high concentrations of environmental pollutants in the reported locations. These findings emphasize the value of dogs as a potential animal model for studying human health and the effect of environmental factors on cancer development.

## Simple Summary

The Global Cancer Observatory (GCO) of the International Agency for Research on Cancer (IARC) has reported a progressive global increase in cancer cases. The same trend is reported among pets, with more than 4.2 million cancer diagnoses in dogs annually. Similar causes or predisposing factors influence cancer development in both humans and animals. Dogs and cats develop tumors with morphological and biological characteristics that closely resemble human cancers, making them valuable translational models for human cancer therapy and sentinels of environmental exposure to carcinogens. Extrapolating veterinary cancer data can provide important information for a comparative approach to oncology and One Health. In this study, we analyzed cases of testicular tumors in humans and dogs, as well as environmental data from cities with testicular tumor occurrences. In these areas, severe contamination of soil and water was recorded. The analysis of oncological and environmental data suggests a strong correlation between the frequency of testicular tumors and the presence of environmental pollutants. These data highlight the potential of comparing cancer frequency and survival patterns between human and animal populations in an area or region over time for determining potential environmental triggers.

## 1. Introduction

The collection and organization of data in the medical field are essential prerequisites for studying complex multifactorial diseases, such as cancer. Both genetic and environmental factors contribute to the disease’s causation, with different expression patterns. Collecting data on neoplastic diseases within a specific population and geographical area is essential to guide targeted actions, determine which groups require intervention, and assess the effectiveness of preventive measures, such as screening programs. Comparative studies on the biological behavior of spontaneous tumors and the possible role played by environmental risk factors can provide valuable insights for preventing neoplasms in humans [1,2,3].

Cancer results from changes in specific genes that alter cells’ function and is often driven by environmental exposures that damage DNA. Not all carcinogenic exposures are avoidable, especially those found in the air, water, food, or everyday materials. Scientists are actively investigating which exposures can cause or contribute to cancer development. Understanding which exposures are harmful and identifying where they occur is essential for reducing risk. Any substance that causes cancer is known as carcinogenic, although not all exposures to a carcinogen will result in cancer. Many factors influence the ability of a person exposed to a carcinogen to develop cancer, including the amount and duration of exposure and the individual’s genetic background. The International Agency for Research on Cancer (IARC) produces regular scientific reports on substances that may increase the risk of cancer in humans. Since 1971, the agency has evaluated more than 1.000 agents, including chemicals, complex mixtures, occupational exposures, physical agents, biological agents, and lifestyle factors. Of these, more than 500 have been identified as carcinogenic, probably carcinogenic, or possibly carcinogenic to humans. Adopting the One Health approach, the World Health Organization (WHO), together with the World Organization for Animal Health (WOAH), emphasizes the importance of considering animal health as integral to the broader picture of global health in which animal welfare is not just a background element [1,2,3].

In the last fifty years, industrialized countries have witnessed a notable rise in the diagnosis of malignant tumors of the breast, prostate, testis, ovary, thyroid, skin, and gastrointestinal tract [4]. Although multiple factors likely contributed to this trend, the rapid increase supports the hypothesis that it may be determined by the exposure to environmental pollutants, especially those capable of interfering with the endocrine system [5,6]. These toxic principles act as endocrine disruptors, heterogeneous substances that can mimic the action of hormones and interact with their receptors, altering their proper stimulation. These substances are included in the large group of persistent organic pollutants (POPs), such as diethylhexyl phthalate (DEHP), polybrominated diphenyl ethers (PBDEs), polychlorinated biphenyls (PCBs), dichloro-diphenyl-trichloroethane (DDT), perfluoroalkyl substances (PFASs), perfluorooctane sulfonate (PFOS), perfluorooctanoic acid (PFOA), perfluorohexane sulfonate (PFHxS), phthalates, bisphenol A (BPA), xenoestrogens (such as Zearalenon), dioxins and furans, heavy metals, aromatics compounds, organotin compounds, hydrocarbons, polycyclic aromatic hydrocarbons, and phytochemicals [7,8,9,10,11,12,13,14,15,16,17,18,19,20,21,22,23,24,25,26,27,28,29,30,31,32,33,34,35]. Testicular tumors, in particular, have received increased attention due to their strong association with endocrine-disrupting chemicals [36]. They are a relatively common cancer in both humans and dogs, and canine seminomas in particular are considered a counterpart of spermatocytic seminomas in men, based on similar age at diagnosis and biological behavior. This makes testicular tumors a compelling subject for comparative research within the One Health framework [37].

### 1.1. Testicular Tumors

The most recent World Health Organization (WHO) classification systems categorize testicular tumors according to their cell of origin into germ cell tumors and sex cord stromal tumors. Testicular germ cell tumors (TGCTs) are further classified into seminomas and non-seminomatous germ cell tumors (NSGCTs), depending on whether the precursor cells are germ stem cells or embryonic epithelial cells, respectively. Both seminomas and NSGCTs originate from precursor lesions known as germ cell neoplasia in situ (GCNIS). In addition, there are non-GCNIS TGCTs, such as spermatocytic tumors, which arise from spermatogonia or spermatocytes. Stromal tumors include interstitial cell tumors (Leydig) and Sertoli (sustentacular) cell tumors (SCTs). In cases where testicular tumors exhibit histological features of both germ cell tumors and stromal cell tumors (usually a combination of seminomas and SCTs in dogs), they are classified as mixed germ cell stromal tumors (MGCSCTs). Other tumor types, such as mesothelioma, fibrosarcoma, or hemangioma, are rarely found in the testes of domestic animals [37].

Several risk factors are involved in the development of testicular tumors in both species, including cryptorchidism, genetic predisposition, congenital anomalies of the testicles, and exposure to environmental pollutants [36,37,38,39,40,41,42]. The incidence and distribution of testicular tumors are different between dogs and humans. In dogs, 24–42% are seminomas, 23–51% interstitial cell tumors or leydigomas, 8–33% Sertoli cell tumors or sertoliomas, and 3–7% mixed germ cell–sex cord stromal tumors. In humans, approximately 50% are seminomas, 50% non-seminomas or mixed TGCTs, 0.4–1.5% Sertoli cell tumors or sertoliomas, 1–3% interstitial cell tumors (leydigomas), and 1% spermatocytic tumors [37,38,39,40,41,42,43].

### 1.2. Environmental Disruptors and Testicular Cancer

Emerging evidence highlights the role of environmental disruptors in the etiology of testicular cancer. In vitro studies have demonstrated that phthalate esters, widely used as plasticizers, may interact with androgen and estrogen receptors, acting as estrogenic and antiandrogenic compounds. Moreover, in vitro and in vivo toxicology studies show that phthalates (Ps) and their metabolites may be associated with congenital abnormalities (cryptorchidism, hypospadias), impaired semen quality, and increased risk of testicular germ cell cancer, confirming the role of these chemicals in disrupting the hormonal balance. Furthermore, Ps interfere with male reproductive system development, impairing the function of Leydig and Sertoli cells. These disruptions contribute to a clinical condition known as testicular dysgenesis syndrome (TDS), from which testicular cancer (TC) can arise [37]. The effect of pesticides and insecticides on the male reproductive system has also been examined, with occupational exposure to these compounds leading to impaired semen quality, reduced seminal volume and percentage of motility, increased seminal pH, abnormal sperm head morphology, and leukocyte concentration. Moreover, pesticides interfere with sex hormone concentrations, decreasing serum luteinizing hormone and testosterone levels [36]. Dichloro-diphenyl-dichloroethylene (p, p’-DDE) represents one of the main pesticides extensively studied for its association with TC. p, p’-DDE, which can accumulate in adipose tissue and is poorly excreted, is a metabolite of dichloro-diphenyl-trichloroethane and a potent androgen receptor antagonist, commonly used as a pesticide until it was banned in the 1970s–1980s. Another organochlorine pesticide widely distributed in the environment is hexachlorobenzene (HCB). HCB is a fungicide with physical properties, such as vapor pressure and water solubility, that make it persistent in the ecosystem and able to bioaccumulate. However, the association between HCB and testicular tumor remains controversial [37]. Finally, TC has also been associated with exposure to chlordane and its derivatives (oxychlordane, trans-nonachlor, cis-nonachlor), organic pollutants used as pesticides and then prohibited in many countries due to their toxicity and persistence. Exposure mainly occurs through ingestion of contaminated food, inhalational or dermal routes, and they can accumulate for a long time in human tissues due to their high lipophilicity.

### 1.3. The Aim of the Study

The aim of this study is to collect and analyze data on environmental pollutants and testicular tumors in both canine and human populations in the Campania region, and to identify frequencies and assess trends in testicular cancer. The study also seeks to investigate the role of environmental contaminants implicated in the pathogenesis of testicular neoplasms, in order to inform public veterinary health aimed at mitigating or eliminating their effects. Findings will be compared with data from human and environmental studies to support an integrated monitoring and prevention strategy within a One Health framework.

## 2. Materials and Methods

### 2.1. Oncological Data

A retrospective study was conducted in the Campania region between 2020 and 2023. For the oncological analysis, data were extracted from the Campania Animal Cancer Registry, including the following official laboratory databases: Laboratory of Anatomical pathology of the Department of Veterinary Medicine and Animal Production of the University of Naples Federico II and Laboratory of Anatomical pathology of Istituto Zooprofilattico Sperimentale del Mezzogiorno (IZSM). These institutions operate under the Regional Regulation 2 February 2021, n°1—Regulations implementing Regional Law n°3 of 11 April 2019 (provisions aimed at promoting and protecting the respect and welfare of pets and preventing straying). Additional data were obtained from the management system of the National Cancer Institute “Fondazione G. Pascale-IRCCS”. A standardized sample submission form was specifically designed for cancer case registration and made available on the laboratory websites. The specimen submission form collected specific animal information, including species, age, sex, breed, castration/spay status, and identification number. The owner’s residential address was also included. Animal cancer data used in this study included species, cancer diagnosis, ICD-O classification, and municipality of origin. Formalin-fixed specimens were routinely processed, included in paraffin, and stained with hematoxylin and eosin (HE) for histologic examination. In the case of poorly differentiated neoplasms, immunohistochemistry was also performed. Tumors were classified according to the latest pet cancer classification systems the of WHO International Histological Classification of Tumors of Domestic Animals (Histological Classification of Tumors of the Genital System of Domestic Animals Vol. IV) and coded according to the canine International Classification of Diseases for Oncology (Vet-ICD-O-Canine-1, a System for Coding Canine Neoplasms Based on the Human ICD-O-3.2) to facilitate comparison with existing human and animal cancer registries [44,45].

### 2.2. Environmental Data

Furthermore, environmental data was obtained from the Ministry of Environment and Energy Security website and the Campania Regional Agency for Environmental Protection (ARPAC). The ministry’s website provided information on Sites of National Interest, where water and soil are officially defined as contaminated and remain under the jurisdiction of the Ministry of the Environment. Part of the environmental data was obtained from the ARPAC website, which classifies the areas into potentially contaminated areas and contaminated areas to be remediated. Contaminated sites identify areas with polluted environmental matrices. A site is defined as potentially contaminated when one or more contamination threshold concentration values (CSCs) defined in Tables 1 and 2 of Annex 5 of part IV title V of Legislative Decree No. 152/2006 have been exceeded in the environmental matrices “soil,” “subsoil,” “landfill materials,” and “groundwater”. On the other hand, a site is defined as contaminated when the threshold concentrations of risk (CSRs), calculated through the site-specific health–environmental risk analysis (Annex 1, part IV, title V of Legislative Decree No. 152/2006), have been exceeded. The environmental data of the municipalities considered refer to the Regional Council resolution no.685 del 30/12/2019 [Official Bulletin of the Campania Region (BURC) n. 3 of 13/01/2020] [46,47,48,49].

### 2.3. Geographic Information Systems

Geographic information systems, or GISs, were used to map dog and human tumors in the Campania region, focusing on municipalities of residence and mapping pollutants in the same region. A GIS is an interconnected set of hardware, software, human, and intellectual components to acquire, store, process, analyze, and display information derived from georeferenced data. A GIS is a valuable tool for integrating data originating from different sources, providing visualization on a map using information in space and time, and allowing the identification of underlying patterns, associations, and trends. The GIS links data to a map, integrating spatial data (location) with descriptive attributes (characteristics). The GIS helps users understand patterns, relationships, and geographic context. Benefits include improved communication and efficiency as well as better management and decision-making. For this study, spatial data were analyzed and visualized using Geographic Information System software (QGIS 3.40.1, General Public License), which allowed us to manage spatial datasets, conduct geospatial analyses, and generate thematic maps, facilitating the integration of oncological and environmental information across the region [50,51,52,53].

### 2.4. Sites of National Interest (SINs)

Sites of National Interest, or SINs, represent large, contaminated areas classified as hazardous by the Italian state and thus require remediation of soil, subsoil, and/or surface and groundwater to prevent environmental and health risks. SINs were defined by the Legislative Decree 22/97 (Ronchi Decree) and Ministerial Decree 471/99 and later incorporated into the Legislative Decree 152/2006. The sites are identified based on their characteristics and the quantity and hazardousness of pollutants, as well as the significance of the impact on human health, ecosystems, and cultural and environmental heritage [53]. National contaminated sites include areas in which human activities previously carried out or in progress have altered the qualitative characteristics of soils, surface, and groundwater and specifically include -disused industrial areas; industrial areas undergoing reconversion; industrial areas in operation; sites affected by asbestos production and mining activities; ports; areas that have been the subject of accidents involving the release of chemical pollutants in the past; former mines, quarries, non-compliant landfills, and illegal dumps. At these sites, exposure to contaminants can occur through occupational exposure, industrial emissions, and contaminated soils and groundwater [53]. The areas monitored by the ministry are divided into the following:

- Sites of National Interest transferred to regional jurisdiction (SIR): Area del Litorale Vesuviano, Litorale Domizio Flegreo, Bacino Idrografico del fiume Sarno, and Pianura.
- Sites of National Interest that remain under the jurisdiction of the Ministry of the Environment (SIN): Napoli Orientale (with quarters of Secondigliano, San Pietro a Patierno, Ponte della Maddalena, Sant’Erasmo, Poggioreale, San Giovanni a Teduccio, Barra, and Ponticelli) and Napoli Bagnoli–Coroglio.
- City of Giuliano: Undergoing perimeter definition, currently transitioning from a SIR to SIN.

### 2.5. Oncological and Environmental Approach

We adopted a dual approach: an environmental approach starting from the identification of the Sites of National Interest (SINs) of the Campania Region (examining the soil and water monitoring) to correlate them with the cancers most present in those areas, and an oncological approach using cancer registry data to highlight areas with a higher frequency of a specific environment-related tumor and then correlate them to pollution-related factors.

### 2.6. Statistical Analysis

GraphPad Prism 8 software version 8.4.3 (GraphPad Software, San Diego, CA, USA) was used for statistical analysis. Statistical differences between the groups were assessed using the Mann–Whitney U test to determine differences in medians, the F-test for variance, and Kolmogorov–Smirnov tests to compare distributions. Values of *p* < 0.05 were considered significant.

### 2.7. Crude Incidence Rate (CIR) and Standardized Incidence Risk (SIR)

To improve the results, we added and compared the crude incidence rate (CIR). We calculated the standardized incidence risk (SIR) of testicular tumors in the dog and human populations with the indirect model. We extrapolated the data concerning the dog reference population and the human population from the regional dog registry system and ISTAT data, respectively [54,55].

## 3. Results

### 3.1. Environmental Approach

**Figure 1:**
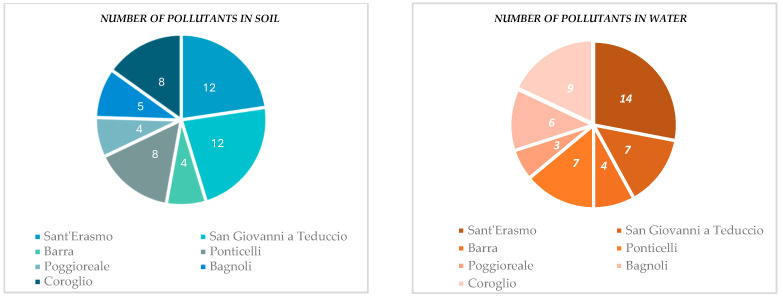
Concentration of pollutants in the SINs of the Campania region.

In SINs, the pollutants present were acetone, carcinogenic chlorinated aliphatics, non-carcinogenic chlorinated aliphatics, aliphatic compounds, ammonia, aromatics, chlorobenzene, organnostanic compounds, dioxin, ethyl-t-butyl ether (ETBE), phenols, phytopharmacies, pesticides, furans, hydrocarbons, PAHs, metals, metalloids, methyl-t-butyl ether (MTBE), and PCBs. The number of pollutants per area within the SINs is visualized in Figure 1.

Types of testicular tumors reported in SINs included the following:

- In dogs:
  Naples (Secondigliano): Testicular tumors of the non-germ cell line (Leydig cell tumors).
  Naples (Poggioreale): Non-germ cell line testicular tumors (Leydig cell tumors).
  Naples (Ponticelli): Testicular tumors of the germ line (seminomas).
- In humans:
  Naples: Germline and non-germ cell line testicular tumors (seminomas, mixed, embryonal carcinoma, liposarcoma, yolk sac teratoma, neuroendocrine tumor, teratoma).
  Naples (Ponticelli): Testicular tumors of the germ cell line (seminomas).
  Naples (Secondigliano): Testicular tumors of the germ cell line (seminomas).

### 3.2. Oncological Approach

**Figure 2:**
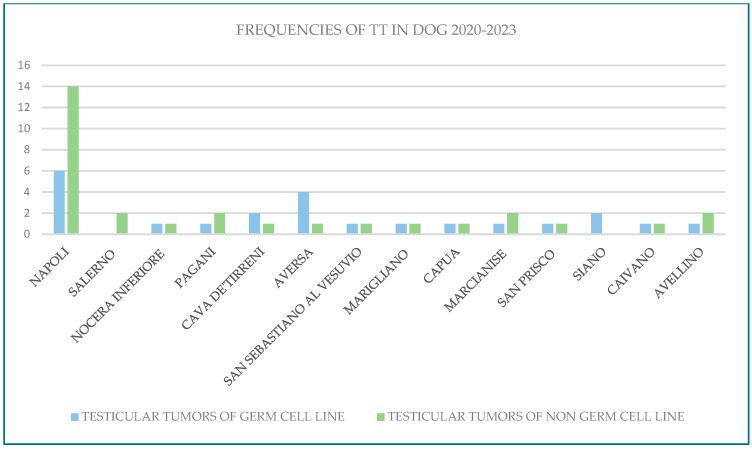
Frequencies of testicular tumors in dogs in 2020–2023.

**Figure 3:**
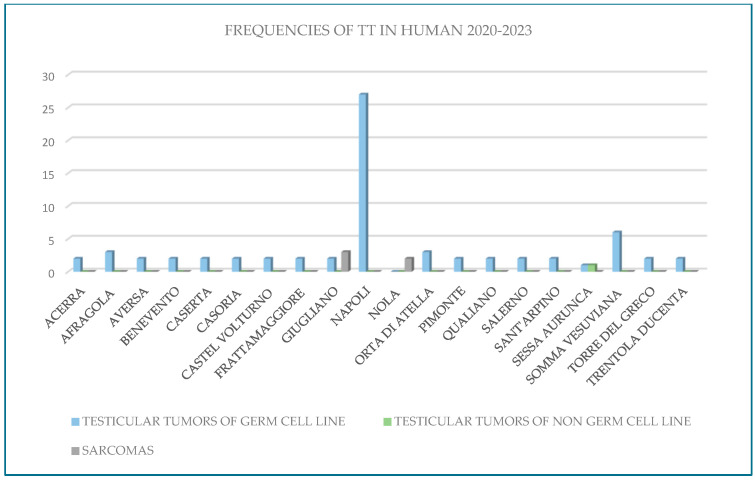
Frequencies of testicular tumors in humans in 2020–2023.

The retrospective analysis of canine testicular oncology data from the Animal Cancer Registry of the Campania region for the years 2020–2023 yielded the following results: 54 testicular tumors in 2020, 65 cases in 2021, 37 in 2022 and 65 testicular tumors in 2023 (Leydig cell tumors, Sertoli cell tumors, and seminomas; Figure 2). For the same period, data from the management system of the National Cancer Institute “Fondazione G. Pascale-IRCCS” revealed 30 cases in 2020, 49 cases in 2021, 51 cases in 2022, and 44 cases in 2023 (mixed tumors and seminomas, with a lower number of Leydig cell tumors and Sertoli cell tumors; Figure 3). In total, 221 canine testicular tumor cases and 174 human testicular tumor cases were reviewed over the four-year study period.

### 3.3. Correlation Between Cancer and Pollutants

**Figure 4:**
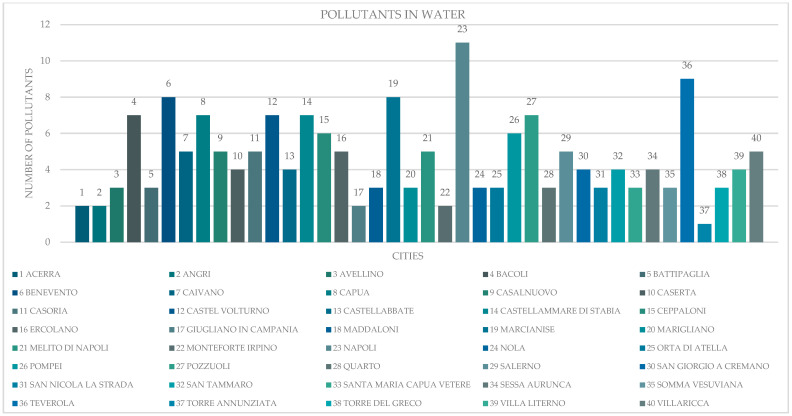
Number of pollutants in the water of the municipalities with high frequencies of testicular tumors.

**Figure 5:**
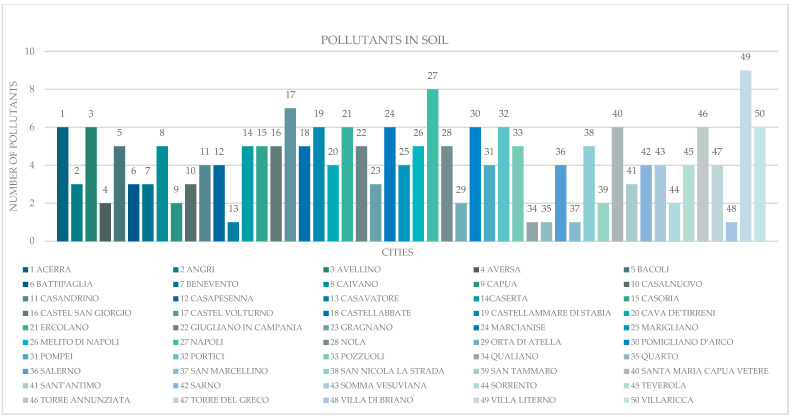
Number of pollutants in the soil of the municipalities with high frequencies of testicular tumors.

**Figure 6:**
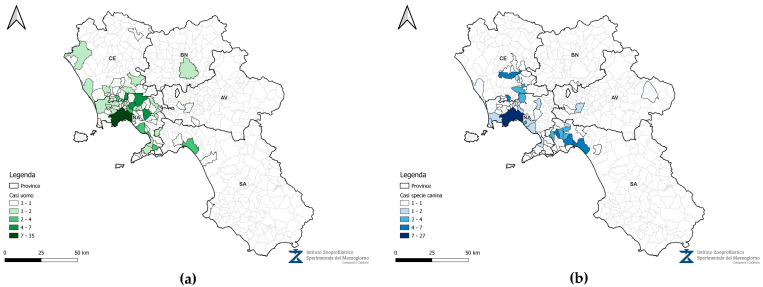
Cartographic representation, using QGIS 3.40.1, of canine (**a**) and human (**b**) tumors in the municipalities of the Campania region.

**Figure 7:**
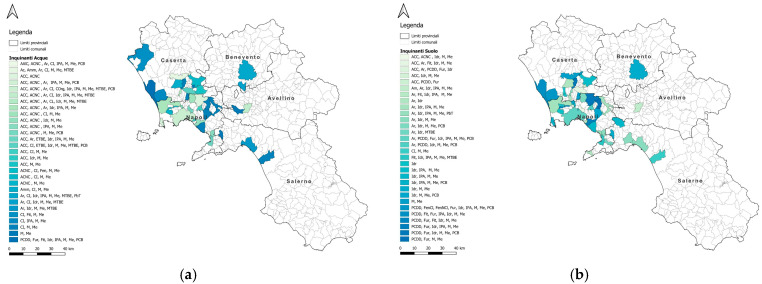
Cartographic representation, using QGIS 3.40.1, of pollutants in water (**a**) and soil (**b**) in the municipalities of the Campania region.

The areas with the highest frequency of testicular tumors in humans and dogs were the municipalities of Napoli and Salerno, and their respective provinces. In the same locations, severe contamination of soil and water by organostannic compounds, dioxin, ethyl-t-butyl ether, phenols, pesticides, furans, hydrocarbons, PAHs, metals, metalloids, methyl-t-butyl ether, and PCBs was recorded [4] (Figure 4 and Figure 5). Using geographic information systems, testicular tumors in dogs and humans showed a high degree of co-localization (Figure 6) consistent with the concentration of pollutants in the soil and water (Figure 7).

In Napoli, San Sebastiano al Vesuvio, Caivano, Marigliano, Acerra, Afragola, Casoria, Frattamaggiore, Giugliano, Nola, Pimonte, Qualiano, Somma Vesuviana and Torre del Greco, the 55 cases of testicular tumors in humans and the 26 cases of testicular tumors in dogs were associated with the presence of metals, metalloids, hydrocarbons, dioxins, furans, PCBs, PAHs, aromatic compounds, carcinogenic chlorinated aliphatics, MTBEs, and organnostanin compounds in water and soil.

In Caserta, Aversa, Capua, Marcianise, San Prisco, Castel Volturno, Orta di Atella, Sant’Arpino, Sessa Aurunca, and Trentola Ducenta, the 15 cases of testicular tumors in humans and the 12 cases of testicular tumors in dogs were associated with the observation of hydrocarbons, aromatic compounds, metals, metalloids, PAHs, MTBEs, ETBEs, dioxins, furans, and PCBs in water and soil.

In Salerno, Nocera Inferiore, Pagani, Cava de’Tirreni, and Siano, the 2 cases of testicular tumors in humans and the 12 cases of testicular tumors in dogs were associated with the finding of metals, metalloids, hydrocarbons, aromatic compounds, and MTBE in water and soil.

### 3.4. Statistical Analysis

**Figure 8:**
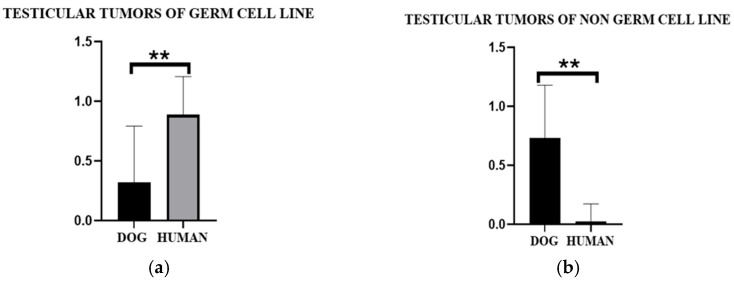
Asterisks represent statistically significant differences between groups in the Mann–Whitney U test to determine differences in medians, the F-test for variance, and the Kolmogorov–Smirnov tests to compare distributions (** *p* < 0.01) for testicular tumors of the germ cell line and testicular tumors of the non-germ cell line in dogs (**a**) and humans (**b**).

To analyze the differences between the canine and human populations regarding testicular tumors, three statistical tests were performed: Mann–Whitney, Kolmogorov–Smirnov, and F-test for variance. The results were evaluated separately for the two types of tumors: germ cell line and non-germ cell line [Figure 8]. Germ cell line tumors are significantly more represented in the human population than in the canine population. The variances of the germ cell tumor data in the two groups are significantly different, with greater dispersion in one of the two species. The cumulative distributions of germ cell tumors in dogs and humans are different, confirming a distinct mode of presentation or frequency of germ cell tumors. Concerning germ cell tumors, a complete inversion is observed: here, the median is significantly higher in dogs, suggesting a greater prevalence or relevance of non-germ cell tumors in the dog population. The distributions between the two species are significantly different, with an even greater divergence in germ cell tumors. The variances are significantly different between dogs and humans, confirming a greater dispersion of values in either group.

### 3.5. Crude Incidence Rate (CIR) and Standardized Incidence Risk (SIR)

From the regional dog registry system and ISTAT data [54,55], we extrapolated the information useful for calculating crude incidence rate and standardized incidence risk (Table 1).

We have calculated and compared the CIR = N/O, respectively:

*CIR Dog* = 512.791/221 = 0.000431

*CIR Human* = 28.876.799/174 = 0.000006025

*CIR Dog/CIR Human* = 0.000431/0.000006025 = 71.5

The crude incidence rate of testicular cancer in dogs in Campania is about 71.5 times higher than in the human male population.

To calculate the standardized incidence risk (*SIR*), we used an indirect standardization model (we did not have access to the age of all dogs):

Expected cases in Dog (*E*) = *CIR Human* × *N Dog*

Expected cases in dogs if they had the same *CIR* as humans:

*E = CIR × N* = 0.000006025 × 512.791 = 3.09

*SIR Dog* = *O/E* = 221/3.09 = 71.5

Dogs have a 71.5 times higher risk of testicular cancer than humans, using the human rate as a reference.

## 4. Discussion

Using an integrated environmental and oncological approach, we initially identified the Sites of National Interest (SINs) in the Campania region by analyzing soil and water monitoring data. We then correlated these areas with the most frequently reported neoplasms, using Cancer Registry data to pinpoint areas exhibiting an increased frequency of specific tumors potentially linked to environmental factors and pollution. This methodology aimed to minimize significant sources of bias, including underestimating the true frequency of canine testicular tumors and the synergistic effects of pollutant mixtures, which may act cooperatively even when individual concentrations do not exceed minimal risk thresholds.

The municipalities of Napoli, Caserta, and Salerno, along with their respective provinces, emerged as the areas with the highest incidence of testicular tumors in both humans and dogs. These same regions also showed substantial soil and water contamination. However, a consistent direct proportionality between the number or type of pollutants and tumor frequency was not observed, likely due to individual susceptibility to carcinogenesis and the variable carcinogenic potential of different substances.

In the city and province of Naples, 55 human and 26 canine testicular tumor cases were recorded, and 11 different pollutants potentially associated with carcinogenesis were identified. Despite being the smallest of the Campania provincial capitals by geographic area, Naples has the highest population density for both humans and dogs. The combination of elevated population density and the diversity and concentration of pollutants positions this area as the most at risk among those analyzed.

In Caserta and its province, 15 human and 12 canine testicular tumor cases were reported, associated with the same number of pollutant types. Although this area covers a larger geographical region than Naples, it has a lower population density. A similar number of pollutants suggests that their concentrations and interactions may be less carcinogenic or that the local population may possess greater resilience to environmental carcinogens.

In Salerno and its province, 2 human and 12 canine testicular tumor cases were identified, associated with five potentially carcinogenic pollutants. Salerno represents the largest geographical area among the Campania provincial capitals and ranks second in population density. Although the number of pollutants identified is approximately half that observed in Naples and Caserta, the incidence of canine testicular tumors exceeds ten cases, suggesting a stronger environmental influence on the canine population due to pollutant concentrations and combinations.

Testicular tumors are comparable across different species, as they are triggered by similar mechanisms and display similar biological behavior. Dogs living in close contact with humans are exposed to common oncogenic factors [2]. With their shorter life cycle, they can serve as early warning systems for the development of spontaneous tumors, with particular attention to tumors linked to environmental pollution [1,2].

As mentioned above, in both species, cryptorchidism, genetic predisposition, congenital anomalies of the testicles, and exposure to environmental pollutants are considered several risk factors for the insurgence of testicular tumors. A large percentage of seminomas in dogs overlap with spermatocytic seminomas in humans, which are rare and unrelated to cryptorchidism. Canine seminomas and spermatocytic seminomas tend to appear later in life, whereas human seminomas mainly affect young men, further denoting differences in tumor behavior between species [37]. One possible explanation for the late onset of seminomas or spermatocytic tumors in dogs could be prolonged exposure to environmental factors necessary for tumorigenic transformation and acknowledging the need for longitudinal studies to support this. The oncoepidemiological approach demonstrated here facilitates the development of new environmental monitoring strategies by leveraging data on animal neoplasms and their sentinel role in ecosystem health.

Cancer is among the leading causes of death in both dogs and humans, whereas it remains relatively rare in wildlife and other domestic animals. Compared with humans, the relatively short latency periods for cancer development offer a unique advantage for studying spontaneous diseases in animal models. However, a significant challenge in environmental epidemiology is the accurate assessment of exposure.

The differences in statistical analysis are significant and could reflect biological, environmental, or genetic peculiarities of the two species, paving the way for further comparative investigations in veterinary and human oncology.

Study data suggest that testicular cancer is more frequent in dogs than in male humans in Campania, with a SIR of 71.5. Dogs are frequently exposed to indoor dust through both oral and dermal routes. Several environmental and physiological factors may account for the increased standardized incidence ratio (SIR) of testicular tumors observed in the canine population relative to humans.

Household dust is a major vector of endocrine-disrupting chemicals (EDCs) such as phthalates, bisphenol A (BPA), polybrominated diphenyl ethers (PBDEs), and per- and polyfluoroalkyl substances (PFASs). These substances have been identified in significant concentrations in indoor environments and have been shown to affect both human and canine endocrine systems. Studies have confirmed that dogs, due to their behavior (e.g., grooming and floor contact), are particularly susceptible to this route of exposure [56].

Commercial dog food has been shown to contain measurable levels of environmental contaminants, including phthalates, PCBs, PBDEs, and BPA, especially in canned products, where concentrations of BPA may reach up to 206 ng/g. Additionally, phytoestrogens from soy and mycotoxins such as zearalenone (ZEA), which exhibit estrogenic activity, are commonly found in dry kibble. These compounds may disrupt endocrine regulation and promote testicular carcinogenesis [39].

Dogs exhibit a reduced capacity to metabolize and eliminate lipophilic compounds, leading to bioaccumulation of EDCs. Several studies have demonstrated that dogs present significantly higher serum and tissue concentrations of PBDEs and phthalates compared to humans, often ranging from 1 to 4.5 times higher. This metabolic limitation increases the internal burden of carcinogenic substances in dogs over time [39].

Plastic materials used in dog toys and accessories, such as chewable toys and bumpers, have been identified as a relevant source of phthalates (e.g., DEHP) and BPA. These substances are released into simulated canine saliva and have been shown to exert antiandrogenic and estrogen-like activities, potentially contributing to hormonal imbalance and tumor development in reproductive organs [57].

These findings demonstrate that dogs, due to their diet, behavior, and metabolic characteristics, experience a cumulative and effective exposure to endocrine-disrupting chemicals (EDCs) potentially leading to testicular tumor development.

## 5. Conclusions

The integrated environmental and oncological investigation in this study suggests a complex relationship between environmental pollution and testicular tumors in the Campania region’s human and canine populations. While areas such as Naples, Caserta, and Salerno exhibited significant environmental contamination and high tumor rates, a linear relationship between pollutant presence and cancer frequency was not consistently observed. This suggests that factors such as individual susceptibility, the synergistic effects of pollutant mixtures, and differences in species-specific tumor behavior play a crucial role in carcinogenesis.

Furthermore, the disparities observed in tumor types and their associations with conditions like cryptorchidism between humans and dogs highlight the complexity of identifying shared etiopathogenetic mechanisms. Nevertheless, this study emphasizes the value of adopting an oncoepidemiological approach, where companion animals serve as effective sentinels for environmental health risks. This methodology enhances environmental monitoring precision and offers a foundation for future longitudinal studies to better understand the long-term impact of environmental exposures on human and animal health in a One Health vision.

The biases of this study are related to the methodology used to collect canine cancer cases and the inherent difficulty in consistently establishing clear cause–effect relationships. Scientific dissemination, public awareness campaigns, and screening initiatives may help expand the dataset of the regional animal cancer registry, thereby strengthening comparative analyses with the human cancer registry. It is not always possible to associate the presence of environmental pollutants, especially when levels do not exceed risk thresholds, with tumor development. Nevertheless, the ability to detect even trace amounts of toxic analytes, when interpreted alongside existing literature, may improve both the understanding and perception of the issue.

## Figures and Tables

**Table 1:** Number of observed cases and populations at risk in the Campania region.

Male Population	Male Observed Cases (O)	Male Populations at Risk (N)
Dog	221	512.791
Human	174	28.876.799

## Data Availability

All relevant data are listed in the manuscript.
